# Association between Berlin questionnaire index and blood pressure, organ damage and metabolic profilein a general population

**DOI:** 10.1111/jch.14586

**Published:** 2022-10-18

**Authors:** Jennifer Vanoli, Raffaella Dell'Oro, Rita Facchetti, Michele Bombelli, Giuseppe Mancia, Guido Grassi

**Affiliations:** ^1^ Clinica Medica, Department of Medicine and Surgery University of Milano‐Bicocca Milan Italy; ^2^ University of Milano‐Bicocca Milan Italy

**Keywords:** ambulatory blood pressure/home blood pressure monitor, risk assessment, sleep problems and hypertension

## Abstract

We evaluated the relationships between Berlin questionnaire (BQ) scores, hypertension and other metabolic variables in 598 subjects (age: 65.8 ± 10 years, mean ± SD) enrolled in the PAMELA (Pressioni Arteriose Monitorate E Loro Associazioni) study representative of the general population, treated or untreated with antihypertensive drugs. Two hundred and eleven subjects (35%) had a positive BQ with two or more positive categories of the inquiry. Compared to those without sleep disorders these subjects showed a greater male prevalence (55.9%), worse serum cholesterol, triglycerides and glucose profile, greater body mass index (BMI) (28.9 ± 4.9 vs. 24.9 ± 3.4 kg/m^2^), higher office (and to a lesser extent 24‐h) BP and HR values, higher serum creatinine values and greater rate of echocardiographic left ventricular (LV) hypertrophy (25% vs. 13%). These differences were not detected when the data analysis was restricted to treated hypertensive patients. Thus, BQ scores allow to identify among subjects belonging to a general population those with elevated BP, organ damage and altered metabolic. When antihypertensive drug treatment is present, however, the approach fails to detect differences between groups with low or high BQ index.

## 1 INTRODUCTION

Berlin questionnaire (BQ) allows to identify a condition of sleep fragmentation and to detect high risk patients for obstructive sleep apnea (OSA), thereby representing a method useful in the clinical settings with limited capabilities to refer or perform direct overnight polysomnographic evaluation.^1^, ^2^, ^3^ OSA is a condition known to be related to the development and progression of the hypertensive state and other cardiovascular disease^4^ and, recently, also the same condition of sleep fragmentation was associated with a high likelihood of accelerated atherosclerosis.^5^ Scarce information is available on whether different scores of the BQ might be associated in a general population with differences in the hemodynamic and metabolic profile, as well as in organ damage, thereby allowing to use this evaluation for the assessment of the global cardiovascular risk profile of a given individual.^6^, ^7^, ^8^ Aim of the present study was to evaluate in a general population sample the relationships between BQ scores and clinic blood pressure, 24‐h blood pressure and blood pressure variability as well. Additional aims were to assess in the same cohort the possible association between BQ index, metabolic variables and organ damage, also evaluating the possible influence of antihypertensive drug treatment on the above‐mentioned relationships.

## 2 MATERIALS AND METHODS

### 2.1 Study population

The PAMELA (Pressioni Arteriose Monitorate E Loro Associazioni) study was carried out in a sample of 3200 subjects representative of the population of Monza (a town near Milan, Italy) for sex and age decades (25–74 years). The participation rate was 64%, allowing to collect data in 2051 subjects. Demographic characteristics of nonparticipants and participants were similar, this being also the case for cardiovascular risk factors as assessed by data collected via phone interviews. From the original sample of 2051 subjects who underwent the initial evaluation in the nineties, we examined for the present cross‐sectional study a sample of 598 survived subjects who attended the third survey in 2016–2017. All variables reported in the present paper were collected during this third survey.

### 2.2 Study variables

Methods employed in the PAMELA study have been previously described in detail elsewhere,^9^ Briefly, after being obtained an informed consent, subjects were invited to undergo a comprehensive clinical evaluation at the outpatient clinic of the Saint Gerardo University Hospital of Monza. Collected data included a comprehensive medical history, a thorough physical examination, body weight and height, standard blood tests, three sphygmomanometric systolic (S) and diastolic (D) BP measurements in the sitting position (office BP), a 12‐lead EKG and an echocardiogram. Body weight was recorded to the nearest .1 kg using a calibrated electronic scale with patients wearing indoor clothing without shoes and body mass index (BMI) was then calculated. Height was recorded to the nearest .5 cm using a standardized wall‐mounted height board. All individuals were then fitted with ambulatory BP monitoring device set to obtain automated oscillometric BP and heart rate (HR) readings every 20 min over the 24 h via a validated ambulatory blood pressure monitoring device (Spacelabs 90207, Issaquah, WA, USA).^9^ During the monitoring period, the individuals were asked to pursue their normal activities.  In each individual, the 24‐h average systolic BP, diastolic BP, and HR average values, standard deviation, cyclic components of variability and individual residual variability were calculated as previously reported.^9^

### 2.3 Measures of end organ damage

For the purposes of the present study assessment of renal function was done via plasma creatinine evaluation (standard assay method) while echocardiography was employed to assess left ventricular mass. The echocardiographic examination was performed according to standardized procedures, as previously reported.^10^ In brief, M‐mode and two‐dimensional echo examinations were carried out with a commercially available instrument (Acuson 128 CF; computer Sonography). End‐diastolic (d) and end‐systolic (s) LV internal diameters (LVID), inter‐ventricular septum thickness (IVS), and posterior wall thickness were measured from two‐dimensionally guided M‐mode tracings recorded at 50–100 cm/s speed, during at least three consecutive cycles. Left ventricular mass (LVM) was estimated by using the corrected American Society of Echocardiography formulae.^11^ Echocardiographic tracings were obtained by two skilled operators and read by a third independent observer. Left ventricular (LV) hypertrophy was alternatively defined when LVMI_BSA_ was equal or higher than 114 grams/m^2^ in men and 99 grams/m^2^ in women, and when LVMI_HT_ was equal or higher than 51 grams/height^2.7^ in men and 47 grams/height^2.7^ in women.^10^

### 2.4 Berlin questionnaire

A BQ completed was available in 598 individuals. Subjects with at least two of three positive categories were considered at high risk for OSA (BQ≥2). Category 1 (snoring assessment) was detected with two or more positive responses among five questions, category 2 (daytime sleepiness) was detected with two or more positive responses among three questions, and category 3 was found if the patient was hypertensive or showed BMI > 30 kg/m^2^.^12^

### 2.5 Statistical analysis

Values were expressed as means ± SD or percentages. Comparisons between subjects with positive BQ (BQ≥2) and negative BQ (BQ < 2) scores were made with Student *t*‐test for unpaired samples or Mann‐Whitney *U*‐test (continuous variables) and with Chi‐Square test (categorical variables). Linear models were used to adjust, by age and sex, the association between BP, HR, metabolic values (dependent variables) and the positive BQ score (independent variable). The same analysis was performed among untreated and treated subjects with antihypertensive drugs. A *p*‐value < .05 was considered to be statistically significant. All analyses were performed using SAS software (version 9.4; SAS Institute Inc., Cary, NC).

## 3 RESULTS

Mean age of the whole population sample amounted to 65.8 ± 10 years and 48.5% was males. Two hundred and eleven subjects (35%) had a positive BQ with two or more positive categories of the inquiry (Table 1). These subjects, when compared to those without sleep disorders, showed a greater male prevalence (55.9%), worse serum glucose and triglycerides profile, greater BMI (28.9 ± 4.9 vs. 24.9 ± 3.4 kg/m^2^), higher office SBP, DBP, and HR values, as reported in Table 1. 24‐h BP also showed a tendency, although not significant, to be greater in this group of subjects, which also displayed significantly greater serum creatinine values and a significantly greater rate of echocardiographic LV hypertrophy (25% vs. 13% for LV mass indexed for height^2^ ^.^ ^7^). The percentage of individuals under antihypertensive therapy was also significantly greater in this group of individuals (72.5% vs. 37.7%). No significant difference between subjects with a BQ < 2 and ≥ 2 was found taking into account blood pressure and heart rate variability data analysis.

**TABLE 1:** Clinical characteristics of the whole study population and of selected subjects with positive (≥2) compared to those with negative (<2) Berlin questionnaire (BQ)

		BQ score		
	Whole group	<2	≥2	*p*‐value
Number	598	387	211	
Age, years	65.8 ± 10	65.4 ± 10.5	66.3 ± 9.2	.2931
Male, %	48.5%	44.4%	55.9%	.0073
BMI, kg/m^2^	26.3 ± 4.4	24.9 ± 3.4	28.9 ± 4.9	<.0001
Office SBP, mmHg	136.3 ± 17.8	134.5 ± 17.7	139.6 ± 17.6	.0008
Office DBP, mmHg	83.3 ± 9	82.7 ± 8.8	84.5 ± 9.3	.0195
Office HR, b/min	70.5 ± 10	69.5 ± 9.5	72.4 ± 10.7	.0012
24 h SBP, mmHg	133.3 ± 13.9	132.6 ± 14	134.6 ± 13.7	.0914
24 h DBP, mmHg	77.9 ± 7.6	77.9 ± 7.5	77.9 ± 8	.986
24 h HR, b/min	72.3 ± 7.5	71.9 ± 7.5	73.1 ± 7.6	.0542
Day SBP, mmHg	138.5 ± 14.6	137.8 ± 14.7	139.7 ± 14.5	.1387
Day DBP, mmHg	81.6 ± 8.4	81.7 ± 8.2	81.5 ± 8.7	.8601
Day HR, b/min	75.7 ± 8	75.3 ± 7.9	76.4 ± 8.3	.1057
SBP night, mmHg	120.1 ± 15.9	119.4 ± 16.2	121.5 ± 15.4	.1237
DBP night, mmHg	68.2 ± 8.6	68.1 ± 8.5	68.4 ± 8.7	.7606
HR night, b/min	63.7 ± 8	63.2 ± 8	64.5 ± 7.8	.0688
SD 24 h SBP, mmHg	21.9 ± 5.9	22 ± 6.2	21.7 ± 5.3	.5634
SD 24 h DBP, mmHg	17.4 ± 5.5	17.4 ± 5.8	17.3 ± 4.9	.6905
SD 24 h HR, b/min	12.6 ± 3.6	12.7 ± 3.4	12.4 ± 3.9	.4045
Residual variability SBP, mmHg	14.1 ± 3.9	14.1 ± 4.1	14 ± 3.4	.6951
Residual variability DBP, mmHg	11 ± 3.9	11.1 ± 4.1	11 ± 3.5	.7759
Residual variability HR, b/min	7.4 ± 2.2	7.5 ± 2	7.3 ± 2.4	.5016
Antihypertensive therapy, %	50.0%	37.7%	72.5%	<.0001
Total cholesterol, mg/dl	200.4 ± 36.2	203.3 ± 35.1	195.1 ± 37.7	.0083
HDL cholesterol, mg/dl	59.2 ± 17	61.9 ± 17.4	54.3 ± 15.1	<.0001
LDL cholesterol, mg/dl	119.9 ± 32.2	121.4 ± 31.8	117.3 ± 32.7	.1384
Triglycerides, mg/dl	107.3 ± 58	101.9 ± 56.3	117.2 ± 59.9	.0003
Glycemia, mg/dl	95.1 ± 21.9	93 ± 20.8	98.9 ± 23.3	<.0001
Creatinine, mg/dl	.92 ± .23	.91 ± .21	.95 ± .26	.0226
LVMI, g/m^2^	85.6 ± 20.6	83.4 ± 20.6	89.7 ± 20.2	.0004
LVMI > 99/114 g/m^2^, %	13.6%	12.9%	15.0%	.4644
LVMI, g/m^2.7^	39.6 ± 10.6	37.8 ± 10.1	43 ± 10.8	<.0001
LVMI > 47/51 g/m^2.7^, %	17.3%	12.9%	25.2%	.0002

*Note*: Data are shown as means ± standard deviation of the mean.

Abbreviations: DBP, diastolic blood pressure; HR, heart rate; LVMI, left ventricular mass index; SBP, systolic blood pressure; SD, standard deviation.

Table 2 shows the analysis of the data according to subgroups of subjects untreated or treated with antihypertensive drugs (beta‐blockers, angiotensin‐converting enzyme inhibitors, angiotensin II receptor blockers, calcium channel blockers, diuretics, alpha blockers in various combinations and daily dosages). While in untreated subjects the differences in the above‐mentioned blood pressure, metabolic and organ damage profile between individuals with and without sleep disorders were confirmed, no significant difference was detected in the group of treated patients, except for a higher rate prevalence of males (58.8% vs. 44.5%) and a greater BMI (28.8 ± 5 vs. 26 ± 3.5 kg/m^2^).

**TABLE 2:** Clinical characteristics of selected subjects with positive (BQ ≥ 2) compared to those with negative Berlin questionnaire (BQ < 2) among subjects untreated and treated with antihypertensive drugs

	No anti‐hypertensive therapy			Anti‐hypertensive therapy		
	BQ < 2	BQ ≥ 2	*p*‐Value	BQ < 2	BQ ≥ 2	*p*‐Value
Number	241	58		146	153	
Age, years	62.5 ± 9.7	63.7 ± 9.5	.3941	70.4 ± 9.9	67.4 ± 8.8	.0059
Male, %	44.4%	48.3%	.5942	44.5%	58.8%	.0134
BMI, kg/m^2^	24.3 ± 3.1	29.1 ± 4.9	<.0001	26 ± 3.5	28.8 ± 5	<.0001
Office SBP, mmHg	131.2 ± 17.2	138.9 ± 18.1	.0030	139.9 ± 17.4	139.9 ± 17.4	.9926
Office DBP, mmHg	82.4 ± 8.2	86 ± 9.8	.0035	83.2 ± 9.7	83.9 ± 9.1	.5275
Office HR, b/min	69.3 ± 8.8	74.1 ± 9.9	.0003	69.9 ± 10.4	71.8 ± 10.9	.1419
24 h SBP, mmHg	131.6 ± 13	137 ± 12.8	.0043	134.3 ± 15.5	133.7 ± 14	.7412
24 h DBP, mmHg	78.4 ± 6.9	79.2 ± 7.2	.4371	76.9 ± 8.2	77.3 ± 8.2	.6405
24 h HR, b/min	72.6 ± 7.4	75.7 ± 7.1	.0045	70.6 ± 7.5	72.1 ± 7.6	.0873
Day SBP, mmHg	137.2 ± 13.8	143.1 ± 13.9	.0041	138.8 ± 16	138.4 ± 14.6	.8219
Day DBP, mmHg	82.4 ± 7.6	83.5 ± 7.9	.3290	80.4 ± 8.9	80.8 ± 8.9	.7258
Day HR, b/min	76.2 ± 7.7	79.2 ± 7.6	.0078	73.8 ± 7.9	75.3 ± 8.3	.1095
SBP night, mmHg	117.1 ± 14.6	121.3 ± 13.8	.0473	123.2 ± 17.8	121.6 ± 16	.4103
DBP night, mmHg	68.2 ± 8.1	68.1 ± 7.6	.9462	68.1 ± 9.2	68.5 ± 9.1	.7347
HR night, b/min	63.5 ± 8.1	66.5 ± 8	.0112	62.7 ± 8	63.7 ± 7.6	.2931
SD 24 h SBP, mmHg	22.2 ± 6.5	22.1 ± 4.4	.9071	21.8 ± 5.7	21.6 ± 5.6	.7607
SD 24 h DBP, mmHg	17.7 ± 6.1	17.7 ± 4.3	.9737	17 ± 5.3	17.1 ± 5.1	.9417
SD 24 h HR, b/min	13.1 ± 3.2	12.8 ± 4	.6163	12 ± 3.7	12.3 ± 3.8	.5953
Residual variability SBP, mmHg	14.1 ± 4.3	14 ± 3.1	.9135	14.2 ± 3.8	14 ± 3.5	.6325
Residual variability DBP, mmHg	11.2 ± 4.3	11.2 ± 3.3	.9759	10.8 ± 3.7	10.9 ± 3.6	.8757
Residual variability HR, b/min	7.7 ± 1.9	7.6 ± 2.5	.9010	7.1 ± 2.2	7.2 ± 2.4	.7174
Total cholesterol, mg/dl	207.7 ± 33.1	203.8 ± 38.8	.4354	196 ± 37.1	191.9 ± 36.9	.3350
HDL cholesterol, mg/dl	63.9 ± 18.4	56.8 ± 17.7	.0090	58.6 ± 15	53.4 ± 13.9	.0022
LDL cholesterol, mg/dl	124.9 ± 30.1	125.8 ± 36.2	.8585	115.6 ± 33.8	114.1 ± 30.9	.6935
Triglycerides, mg/dl	97.5 ± 60	105.4 ± 48	.1320	109.1 ± 49	121.6 ± 63.4	.0839
Glycemia, mg/dl	89.5 ± 16.2	94.4 ± 17.5	.0128	98.7 ± 25.7	100.6 ± 25	.2211
Creatinine, mg/dl	.89 ± .19	.9 ± .23	.6970	.94 ± .24	.97 ± .26	.2156
LVMI, g/m^2^	78.4 ± 16.8	83.6 ± 18.4	.0433	91.7 ± 23.6	92.1 ± 20.4	.8826
LVMI > 99/114 g/m^2^, %	6.4%	10.5%	.2650	23.7%	16.8%	.1410
LVMI, g/m^2.7^	34.9 ± 8	40.6 ± 10.7	.0004	42.6 ± 11.2	43.8 ± 10.7	.3352
LVMI > 47/51 g/m^2.7^, %	6.0%	21.1%	.0003	24.5%	26.8%	.6434

*Note*: Data are shown as means ± standard deviation of the mean. For symbols and abbreviations see Table 1.

Abbreviations: DBP, diastolic blood pressure; HR, heart rate; LVMI, left ventricular mass index; SBP, systolic blood pressure; SD, standard deviation.

Finally, in the whole population, the positive BQ was associated to worse BP, HR, and metabolic values independently by age and sex. In untreated subjects, results were similar except for glycemia and triglycerides (Table 3).

**TABLE 3:** Linear regression models in the whole study population and in subjects untreated with antihypertensive drugs

Whole study population				
Dependent variable	Independent variable	Beta	SE	*p*‐Value
Office SBP, mmHg	BQ > 2	3.80405	1.4113	.0072
Office DBP, mmHg	BQ > 2	1.55439	.76839	.0435
Office HR, b/min	BQ > 2	3.21212	.8429	.0002
Glycemia, mg/dl	BQ > 2	4.42566	1.78712	.0135
Triglycerides, mg/dl	BQ > 2	13.92339	4.95792	.0051
Creatinine, mg/dl	BQ > 2	.01453	.01571	.3554
LVMI, g/m^2^	BQ > 2	4.10488	1.54731	.0082
LVMI, g/m^2.7^	BQ > 2	4.40913	.80256	<.0001
Untreated subjects				
Office SBP, mmHg	BQ > 2	6.56002	2.3085	.0048
Office DBP, mmHg	BQ > 2	3.46199	1.22183	.0049
Office HR, b/min	BQ > 2	4.87586	1.30612	.0002
Glycemia, mg/dl	BQ > 2	4.34156	2.3126	.0615
Triglycerides, mg/dl	BQ > 2	6.93661	8.40039	.4096
Creatinine, mg/dl	BQ > 2	‐.00032	.02227	.9885
LVMI, g/m^2^	BQ > 2	3.58495	2.10888	.0902
LVMI, g/m^2.7^	BQ > 2	4.96946	1.1144	<.0001

*Note*: Symbols and abbreviations as in the preceding tables. All models are adjusted for age and sex.

Abbreviations: DBP, diastolic blood pressure; HR, heart rate; LVMI, left ventricular mass index; SBP, systolic blood pressure.

## 4 DISCUSSION

BQ allows to identify a condition of sleep fragmentation and to detect high risk patients for OSA, both conditions related to the development of cardiovascular disease, thereby representing a method useful in the settings with limited capabilities to refer or perform direct overnight polysomnographic evaluation made for the OSA's diagnosis. Scarce information is available on whether different scores of the BQ might be associated in a general population with differences in the hemodynamic and metabolic profile. Only one previous study has described the association between BQ index and lipidic variables in a general population,^6^ while the other studies available in the literature were mainly focused on the diagnostic accuracy of BQ in specific population, that is, affected by diabetes or metabolic syndrome.^7^, ^8^

The present study provides two sets of new information on the association between BQ score and hemodynamic, metabolic and organ damage profile in a general population. First, our data, collected in a quite large population sample, show that BQ allows to detect, in a general population, the subjects characterized by a worse hemodynamic and metabolic assessment, as defined on the basis of higher office and 24‐h blood pressure and heart rate values, more marked metabolic alterations and more pronounced cardiac and renal organ damage. Second, the sensitivity of the BQ approach to detect high risk patients appears to be reduced in hypertensive patients under antihypertensive drug treatment. Our study does not allow to clarify the reasons responsible for this finding. We can hypothesize, however, that under antihypertensive treatment, particularly if effective as the one employed in treating hypertensive patients of the PAMELA cohort, several hemodynamic, metabolic and organ damage alterations are favorably affected by antihypertensive drugs, which thus decrease blood pressure to normal or near normal values and reverse the metabolic and cardiovascular structural alterations found in the patients.^13^ Additional results of the study are worthy to be mentioned. These include the finding that (1) an increased BQ index is capable to detect individuals displaying greater plasma cholesterol, triglycerides and glucose levels, thereby closely mirroring the main metabolic alterations frequently accompanying OSA, (2) blood pressure variability does not appear to be associated with different BQ indices, (3) day/night blood pressure profile also appears to be unrelated to BQ, and (4) BQ indices ≥2 are associated with more elevated office and 24‐h heart rate values. Given the close association between elevated heart rate and the adrenergic overdrive,^14^ this finding may suggest that an increased BQ index is associated with augmented sympathetic neural influences on the heart and the peripheral circulation, as a result of the marked stimulation OSA may exert on the central and peripheral adrenergic neural drive.^15^

## 5 STUDY LIMITATIONS

Our study has some limitations. The first limitation refers to the cross‐sectional nature of the study's findings, which prevented the possibility to assess whether and to what extent the BQ index might be able to detect in a dynamic fashion the changes in the hemodynamic, metabolic, organ damage and risk profile of a given subject occurring throughout months or years. The second limitation concerns the limited sample size of the subgroups of subjects with different BQ indices and untreated or treated hypertension. Another limitation of our study is related to the survival bias: the original sample was representative of the general population of Monza but our study population represents a subgroup of this, that is, survivors at a 25‐years of follow‐up, and thereby likely presenting a lower CV risk.

## 6 CONCLUSIONS

The results of the present study provide evidence on the clinical utility of the BQ index evaluation for detecting the presence not only of OSA and OSA‐related sleep fragmentation but also for allowing to identify among subjects belonging to a general population those with elevated BP, organ damage and altered metabolic profile. When antihypertensive drug treatment is present, however, the approach fails to detect differences between groups with low or high BQ index.

## CONFLICT OF INTEREST

The authors declare that they have no conflict of interest.

## AUTHOR CONTRIBUTIONS

Jennifer Vanoli and Michele Bombelli collected the data, Raffaella Dell'Oro blindly analyzed the data, Rita Facchetti made statistical analysis and Guido Grassi and Giuseppe Mancia wrote the first draft and the revised versions of the paper, after comments and criticisms by coauthors.

## PATIENT CONSENT STATEMENT

Written informed consent was obtained from all study participants or from their next of kin.

## PERMISSION TO REPRODUCE MATERIAL

Not required.

## CLINICAL TRAIL REGISTARTION

Not required.
